# The role of indigenous knowledge in advancing the therapeutic use of medicinal plants: challenges and opportunities

**DOI:** 10.1080/15592324.2024.2439255

**Published:** 2024-12-09

**Authors:** Esther Ugo Alum

**Affiliations:** Department of Research and Publications, Kampala International University, Kampala, Uganda

**Keywords:** Indigenous knowledge, medicinal plants, phytotherapy, biopiracy, ethnopharmacology

The current therapeutic use of medicinal plants is mostly attributed to indigenous knowledge, a system of knowledge that has been transmitted successively over generations.^1^ In a similar vein, traditional healers and herbalists have long employed their expertise of locally obtainable herbs to treat various ailments. This repository of information, developed over millennia, underpins numerous contemporary therapies.^2^ Given the increasing global interest in natural products and phytotherapy, this information is progressively being integrated into the official health systems. However, the effort to connect indigenous knowledge with the existing scientific understanding can be seen as creating extensive opportunities and presenting significant challenges.

The traditional cultures of many nations have employed plants as therapeutics for a wide range of ailments, spanning from a basic cold to a complex chronic condition.^3,4^ Knowledge of this nature is a significant characteristic that is transmitted from one generation to another and encompasses a wide range of health criteria, including physical, spiritual, and environmental dimensions.^5^

Traditional healing procedures across cultures adopt a comprehensive approach that transcends the simple application of medicinal plants. Shamanic medicine encompasses intricate belief systems and psychosomatic influences, targeting both physical and existential dimensions of health.^6^

Healing methods in rural Mexico embody a Mesoamerican perspective that addresses both physical and spiritual well-being, ensuring holistic patient care.^7^ The application of medicinal plants in ancestral healthcare systems frequently incorporates faith-based rituals and blessings, seeking to heal from a biological and holistic standpoint that encompasses the body, soul, spirit, and environment.^8^

Originating from Ayurveda, Traditional Chinese Medicine, and African tribal healers, traditional medicinal plants such as *Curcuma longa* (turmeric), *Azadirachta indica* (neem), and *Panax ginseng* (ginseng) have gained global recognition and have been the foundation of contemporary medications.^9^

Scientific standardization of medicinal plants begins with ethnopharmacological surveys that document traditional practices and assist researchers in identifying specific compounds with pharmacological properties. For instance, the concept of producing the very potent antimalarial medication artemisinin from the plant *Artemisia annua* was derived from a traditional medicine.^10^ Such instances provide evidence for the significance of indigenous knowledge as an integral component to the process of drug discovery and development.

However, the endeavors to integrate indigenous knowledge systems into the contemporary healthcare system face certain obstacles. Recent research underscores the persistent issues of biopiracy and the epistemology surrounding indigenous knowledge in the utilization of therapeutic plants. Biopiracy refers to the exploitation of knowledge and biological resources of tribes by firms in the pharmaceutical industry or scientists without providing proper compensation to the individuals who have been responsible for developing this information.^11^ Colonial legacies that ignore or exploit indigenous knowledge form the foundation of biopiracy.^12^ The pharmaceutical industry’s strategy for bioprospecting exemplifies industrial-capitalist rationality, in opposition to indigenous understandings of nature.^13^ Biopiracy claims challenge intellectual property rights and raise questions about who owns genetic resources and traditional knowledge and how they should be shared fairly.^14^ Consequently, advocates of ancestral communities have called for the improvement of safety mechanisms, including the establishment of stronger intellectual property rights and the implementation of benefit-sharing measures.

The second issue is the deficiency of epistemology characteristic of the indigenous knowledge systems and scientific frameworks employed in research. The reason for this is that while indigenous knowledge includes qualitative study that takes into account the totality while being tailored to a certain area, contemporary scientific research emphasizes quantitative, reductionist research that can be universally applied.^15^ This requires not just collaboration and research harmonization across several fields of study but also acknowledgment of the epistemological aspects in each respective topic.

According to Gebara et al.,^16^ the growing bioeconomy plan in the Brazilian Amazon could lead to epistemic injustice and bioepistemicide, which could make colonial ideas about nature and traditional knowledge stronger. Thus, we need to go beyond narrow definitions of biopiracy and adopt epistemic pluralism to better include ancestral knowledge in the bioeconomy in order to solve these issues. These difficulties highlight the necessity for a more egalitarian methodology in the application of traditional knowledge for therapeutic uses of medicinal plants.

Despite the importance of ancestral knowledge, its oral transmission poses a challenge, making it vulnerable to erosion over time. This knowledge is essential for preserving indigenous culture and rights and guiding environmental management.^17^ Furthermore, contemporary globalization, urbanization, and the erosion of native languages pose significant challenges to indigenous knowledge systems. As young people mature and relocate to metropolitan areas and other contemporary communities, it becomes challenging for the traditional knowledge to be transmitted to the next generation, therefore eliminating itself from indigenous medical practices.^18^ Again, the shift to contemporary health care systems has been accompanied by the depletion of a substantial quantity of knowledge concerning the healing qualities of plants. At present, the diversity of species is decreasing rapidly due to reasons such as habitat degradation, climate change, and unsustainable practices like overharvesting, which pose a threat to the plants used in traditional medicine.^19^ Notwithstanding these difficulties, there exist quite many possibilities to integrate indigenous knowledge in the advancement of the therapeutic applications of medicinal plants. Through collaboration with indigenous communities or integrating local ethnopharmacological data into scientific study, it becomes feasible to discover potentially valuable avenues in the quest for novel pharmaceuticals.^20^ It is important to acknowledge that these collaborations help to establish long-lasting opportunities for the development of indigenous people without eliminating their cultural heritage. Reverse pharmacology is of utmost importance. Reverse pharmacology is a method that involves identifying traditional knowledge and then supporting it with rigorous scientific analyses to confirm the safety and efficacy of medicinal herbs.^20^ Furthermore, ethnopharmacology is an emerging field under ethnobiology that encompasses ethnobotany, pharmacology, and anthropology to comprehend traditional therapeutic systems. Ethnopharmacology is a scientific study of the traditional utilization of plants and other naturally occurring compounds by people for therapeutic purposes. The primary objective of ethnopharmacology is to discover new chemicals sourced from animals and plants for application in traditional medical systems.^20^

Moreover, ethnopharmacological research not only validates the effectiveness of the traditional healers’ knowledge but also offers supplementary insights into potential novel treatment approaches that can be influenced by indigenous knowledge.^21^ Additionally, the deployment of digital technologies has introduced novel methods for documenting and preserving ancestral knowledge. The current electronic databases and archives, which contain a diverse range of material on traditional medicine, are valuable resources for both scientists and indigenous communities. They facilitate the sharing of knowledge while preserving traditional practices.^22^

Similarly, the increasing priority for natural products worldwide and the growing popularity of herbal items present an opportunity to develop a sustainable and reasonably fair system for producing and marketing therapeutic plants. By promoting the expansion of enterprises in the provision of medicinal plants, together with equitable compensation for individuals who contribute their expertise on the geographical distribution of these plants, a market is established to foster the preservation of traditional knowledge and the sustainable utilization of the plants.^23^ For the optimal utilization of indigenous knowledge in the application of plants for disease treatment, it is necessary to employ more than one approach. Efforts should include strengthening collaborations among indigenous communities, scientists, and policymakers, financing scholarly research to assess the safety and efficacy of plants, establishing patent protection legislation, promoting plant cultivation, addressing stable markets, and integrating indigenous knowledge into mainstream healthcare systems. Enabling wider access to natural therapies will also facilitate the preservation of local knowledge.

Altogether, the role of indigenous knowledge in promoting the therapeutic benefits of the plants is significant and convoluted. Challenges such as biopiracy, epistemological disparities, and diminished traditional knowledge must not be disregarded. By acknowledging and incorporating indigenous knowledge into the current scientific discussions, it is feasible to uncover novel treatment opportunities and further the comprehensive approach to the global healthcare system. Furthermore, it is essential to recognize that the preservation and dissemination of indigenous knowledge are not only scientific endeavors but also moral responsibilities focused on the benefit of the people. Future study should prioritize the utilization of traditional knowledge, the examination of plant-human interactions, and the integration of several scientific disciplines to fully harness the potential of medicinal plants.

## Data Availability

Additional data shall be made available by the author on request.
